# The ATP-binding cassette (ABC) transporter OsABCG3 is essential for pollen development in rice

**DOI:** 10.1186/s12284-018-0248-8

**Published:** 2018-10-11

**Authors:** Zhenyi Chang, Mingna Jin, Wei Yan, Hui Chen, Shijun Qiu, Shan Fu, Jixing Xia, Yuchen Liu, Zhufeng Chen, Jianxin Wu, Xiaoyan Tang

**Affiliations:** 1 Guangdong Provincial Key Laboratory of Biotechnology for Plant Development, School of Life Sciences, South China Normal University, Guangzhou, 510631 China; 2 Shenzhen Institute of Molecular Crop Design, Shenzhen, 518107 China; 3 School of Life Sciences, Capital Normal University, Beijing, 10048 China; 4 State Key Laboratory of Conservation and Utilization of Subtropical Agro-bioresources, College of Life Science and Technology, Guangxi University, Nanning, 53004 China

**Keywords:** Rice, Anther, Tapetum, Pollen wall, Exine, Nexine, Intine, ATP-binding cassette (ABC) transporter

## Abstract

**Background:**

The pollen wall, which protects male gametophyte against various stresses and facilitates pollination, is essential for successful reproduction in flowering plants. The pollen wall consists of gametophyte-derived intine and sporophyte-derived exine. From outside to inside of exine are tectum, bacula, nexine I and nexine II layers. How these structural layers are formed has been under extensive studies, but the molecular mechanisms remain obscure.

**Results:**

Here we identified two *osabcg3* allelic mutants and demonstrated that *OsABCG3* was required for pollen development in rice. *OsABCG3* encodes a half-size ABCG transporter localized on the plasma membrane. It was mainly expressed in anther when exine started to form. Loss-function of *OsABCG3* caused abnormal degradation of the tapetum. The mutant pollen lacked the nexine II and intine layers, and shriveled without cytoplasm. The expression of some genes required for pollen wall formation was examined in *osabcg3* mutants. The mutation did not alter the expression of the regulatory genes and lipid metabolism genes, but altered the expression of lipid transport genes.

**Conclusions:**

Base on the genetic and cytological analyses, *OsABCG3* was proposed to transport the tapetum-produced materials essential for pollen wall formation. This study provided a new perspective to the genetic regulation of pollen wall development.

**Electronic supplementary material:**

The online version of this article (10.1186/s12284-018-0248-8) contains supplementary material, which is available to authorized users.

## Background

Pollen development is essential for successful reproduction in flowering plants. Anther is a male reproductive organ with a wall structure enclosing a locule where microspores develop (Zhang et al. 2011). The anther wall consists of four layers of cells, from outside to inside, the epidermis, endothesium, middle layer and tapetum (Zhang et al. 2011). The out surface of epidermis is covered with a cuticle layer of delicate patterns that protects the anther from environmental stresses (Scott et al. 2004; Zhang et al. 2016). The tapetum nourishes the microspore development and secretes materials for pollen wall formation. The pollen wall is important for pollen function as it protects male gametophytes from various environmental stresses and facilitates pollination (Piffanelli et al. 1998; Scott et al. 2004).

The pollen wall surrounding a mature male gametophyte generally has two structural layers: the inner intine and the outer exine (Ariizumi and Toriyama, 2011). The intine is believed to be formed by the male gametophyte, and consists of cellulose, hemicellulose, and pectic polymers similar to the primary walls of common plant cells (Ariizumi and Toriyama, 2011). The building blocks for the exine are believed to be provided by the tapetum (Piffanelli et al. 1998). Despite the surface pattern of the exine is highly diverse across species, the fundamental structure of the exine is highly similar among taxa (Blackmore et al. 2007). The exine commonly comprises two layers, the outer sexine and the inner nexine. The sexine contains an outermost roof, the tectum, and radially directed rods, the bacula (Ariizumi and Toriyama 2011; Shi et al. 2015). These two portions sculpt the species-specific pattern of pollen grains. The nexine consists of layers of nexine I, on which the bacula is anchored, and nexine II, that is laid down on the intine (Ariizumi and Toriyama 2011; Shi et al. 2015). The spaces between the tectum and nexine I are usually filled with the tryphine, hydrophatic materials derived from the tapetum degeneration (Ariizumi and Toriyama, 2011).

Exine development is initiated through the formation of primexine around the distinct haploid microspores in the tetrad covered by the callose wall (Paxson-Sowders et al. 2001). The primexine functions as receptor for initial depositions of sporopollenin precursors that form the proexine structure as the basis of bacula and tectum (Ariizumi and Toriyama, 2011). After dissolution of the callose wall by the tapetum-secreted callases, the released monocellular microspore shows a considerable increase in exine thickness, which is correlated with increased deposition and polymerization of sporopollenin precursors (Blackmore et al. 2007; Ariizumi et al. 2008; Wan et al. 2011). Additional deposition and polymerization of sporopollenin continue till the bicellular pollen stage prior to second mitosis (Ariizumi and Toriyama 2011; Shi et al. 2015; Zhang et al. 2016). In rice plant, the mature exine structure is visually completed, but the intine structure is not clearly visible at the bicellular stage (Shi et al. 2015; Zhang et al. 2016). Subsequently, second mitosis generates tricellular pollen, the intine is formed by the male gametophyte, the tryphine is deposited on the microspore, and nutrients, such as starch and lipid, are accumulated till pollen maturation (Shi et al. 2015; Zhang et al. 2016).

The tapetum plays a pivotal role in pollen wall development by secreting sporopollenin precursors onto the pollen surface (Piffanelli et al. 1998; Scott et al. 2004). Tapetal cells accumulate lipidic molecules in sub-organelles, such as endoplasmic reticulum-derived tapetosomes and plastid-derived elaioplasts (Liu and Fan 2013). In plants such as Arabidopsis, these sub-organelles are released upon degradation of the tapetum, and their contents are deposited to the pollen surface (Hsieh and Huang 2005). In rice and other Poaceae plants, the tapetal cells form Ubisch body, a specialized structure located on the inner side of the tapetum, that carries sporopollenin precursors to the developing microspores (Shi et al. 2015; Zhang et al. 2016). Nonetheless, degradation of tapetum is still crucially important for pollen wall formation in rice. Premature or delayed tapetal degeneration usually results in abnormal pollen development and male sterility (Liu and Fan 2013; Shi et al. 2015; Zhang et al. 2016). Transcription factors reported to be associated with tapetal function and degeneration, such as *DYSFUNCTIONAL TAPETUM 1* (*DYT1*) (Zhang et al. 2006), *DEFECTIVE in TAPETAL DEVELOPMENT and FUNCTION 1* (*TDF1*) (Zhu et al. 2008), *ABORTED MICROSPORES* (*AMS*) (Sorensen et al. 2003) and *AtMYB103/MS188* (Higginson et al. 2003; Zhang et al. 2007) in Arabidopsis, as well as *GAMYB* (Aya et al. 2009), *TAPETUM DEGENERATION RETARDATION* (*TDR*) (Li et al. 2006; Zhang et al. 2008) and *ETERNAL TAPETUM 1* (*EAT1*) (Ji et al. 2013; Niu et al. 2013b) in rice, regulate pollen wall related-genes directly or indirectly. Mutants of these genes have defective tapetum development and pollen exine formation.

Sporopollenin is complex biopolymers mainly consisting of polyhydroxylated aliphatic compounds and phenolics conjugated by ether and ester bonds (Jiang et al. 2013; Shi et al. 2015; Zhang et al. 2016). Genetic approaches have revealed a number of enzymes and proteins required for sporopollenin biosynthesis and deposition, providing clues for the constituents of sporopollenin and mechanism of exine development (Jiang et al. 2013; Shi et al. 2015). Genes involved in aliphatic lipid biosynthesis, such as *MALE STERILITY 2* (*MS2*) (Chen et al. 2011b), *CYP703A2* (Morant et al. 2007)*, CYP704B1* (Dobritsa et al. 2009)*, ACYL-COA SYNTHASE 5 (ACOS5)* (de Azevedo Souza et al. 2009) in Arabidopsis*,* and their orthologs *DEFECTIVE POLLEN WALL* (*DPW*) (Shi et al. 2011), *OsCYP703A3* (Yang et al. 2014)*, OsCYP704B2* (Li et al. 2010a)*, OsACOS12* (Li et al. 2016) in rice, as well as genes in the aromatic lipids/phenolics biosynthesis pathway, such as *POLYKETIDE SYNTHASE A/B* (*PKSA/B*) (Dobritsa et al. 2010; Kim et al. 2010), *TETRAKETIDE TETRAKE REDUCTASE 1/2* (*TKPR1/2*) (Grienenberger et al. 2010) in Arabidopsis and *OsPKS1/2* (Wang et al. 2013; Zhu et al. 2017; Zou et al. 2018) and *DEFECTIVE POLLEN WALL 2* (*DPW2*) (Xu et al. 2017) in rice, have been showed to be essential for sporopollenin precursor synthesis. Some null mutants of these genes in rice also exhibit defective anther cuticle, supporting the view that anther cuticle and pollen wall share common metabolic pathways (Shi et al. 2015).

While the understanding of sporopollenin biosynthesis has advanced rapidly, the studies on transport of sporopollenin precursors from the tapetum to the pollen wall also have significant progress. The large superfamily ATP-binding cassette (ABC) transporter proteins are involved in translocation of a broad range of substances across membranes using energy from ATP hydrolysis (Verrier et al. 2008; Do et al. 2017). In the past few years, several plant ABCG proteins, such as *AtABCG11* (Panikashvili et al. 2010), *AtABCG26* (Quilichini et al. 2010; Quilichini et al. 2014), *AtABCG9*, *AtABCG31* (Choi et al. 2014), *AtABCG1* and *AtABCG16* (Yadav et al. 2014) in Arabidopsis, and *OsABCG26* (Chang et al. 2016b; Zhao et al. 2015) and *OsABCG15* (Qin et al. 2013; Niu et al. 2013a; Zhu et al. 2013; Wu et al. 2014) in rice, have been shown to contribute to pollen wall development. In Arabidopsis, AtABCG11 is required for cutin and suberin formation in reproductive organs and roots development (Panikashvili et al. 2010). AtABCG26 transfers both lipid precursors and polyketides for exine formation (Quilichini et al. 2014). ABCG9 and ABCG31 contribute to the accumulation of steryl glycoside or related compounds on the pollen surface (Choi et al. 2014). *OsABCG15* and *OsABCG26* have different expression patterns from their orthologs *AtABCG26* and *AtABCG11*, and are proposed to act coordinately in transferring sporopollenin precursors from the tapetum for pollen exine and anther cuticle formation (Zhao et al. 2015). However, the chemical nature of the substrates transported by these ABC transporters remains largely unknown.

The complicate process of pollen development and the complex composition of pollen wall constituents implicate that additional ABCG transporters may be involved in translocation of materials from the tapetum to pollen wall. Here, we reported the isolation of two allelic rice male sterile mutants on the *OsABCG3* gene. Morphological and ultrastructural analyses of *osabcg3* plants revealed that the mutant pollen wall contained the sexine and nexine I layers but lacked the nexine II and intine layers. It is apparent that *OsABCG3* regulates the pollen wall development via a mechanism that is different from *OsABCG15* and *OsABCG26*.

## Results

### Isolation and genetic analysis of the male sterile mutant

**Fig. 1:**
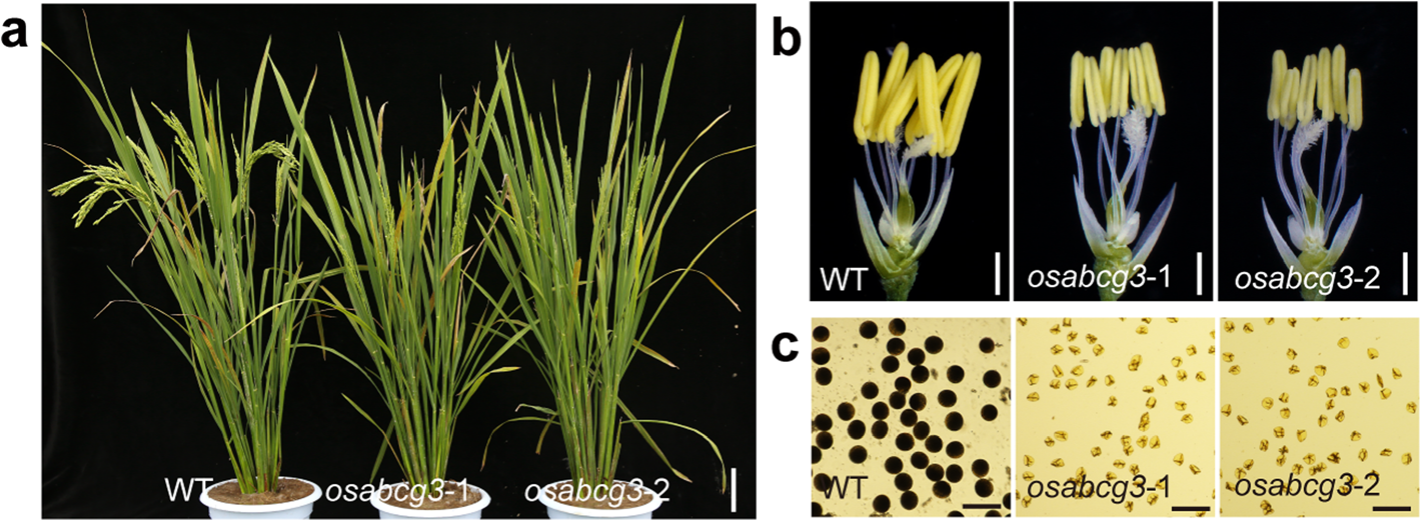
Phenotype of *osabcg3–*1 and *osabcg3–*2. **a** Plants of wild-type (WT), *osabcg3–*1 and *osabcg3–*2 after heading. **b** Spikelets of WT, *osabcg3–*1 and *osabcg3–*2 with the palea and lemma removed. **c** Pollen grains of WT, *osabcg3–*1 and *osabcg3–*2 with I_2_-KI staining. Scale bars = 10 cm (**a**); 1 mm (**b**);100 μm (**c**)

From our rice ethyl methanesulfonate (EMS) mutant library (Chen et al. 2014), two complete sterile mutants were isolated and were designated as *osabcg3*–1 and *osabcg3*–2, because point mutations were revealed in the gene *OsABCG3* (see below). The vegetative growth and flower organ development of these mutants exhibited no obvious differences from that of the wild-type (WT), except the mutant anther being smaller and light yellow (Fig. 1a, b). In order to explain the sterility of these mutants, we investigated the pollen morphology and female fertility. The WT pollen grains were spherical and deeply stained by I_2_-KI, whereas the mutant pollen grains were collapsed and not stained (Fig. 1c). The *osabcg3*–1 and *osabcg3*–2 mutant plants were manually pollinated with the WT pollen. The seed set rates of pollinated *osabcg3*–1 and *osabcg3*–2 were comparable to that of *osnp1*–1 (Chang et al. 2016a), a male sterility mutant without any defects in female reproduction (Additional file 1: Figure S1), indicating that the female fertility of *osabcg3*–1 and *osabcg3*–2 was normal. These results demonstrated that pollen abortion was accountable for the sterility of *osabcg3*–1 and *osabcg3*–2. When *osabcg3*–1 and *osabcg3*–2 were back-crossed with the WT, all F1 progeny were fertile, and the F2 plants displayed an approximate 3:1 segregation of fertile to sterile (301:93 and 259:81 for *osabcg3*–1 and *osabcg3*–2 respectively), suggesting that the sterile phenotypes were sporophytic and controlled by a single recessive gene.

### Cloning of *OsABCG3*

**Fig. 2:**
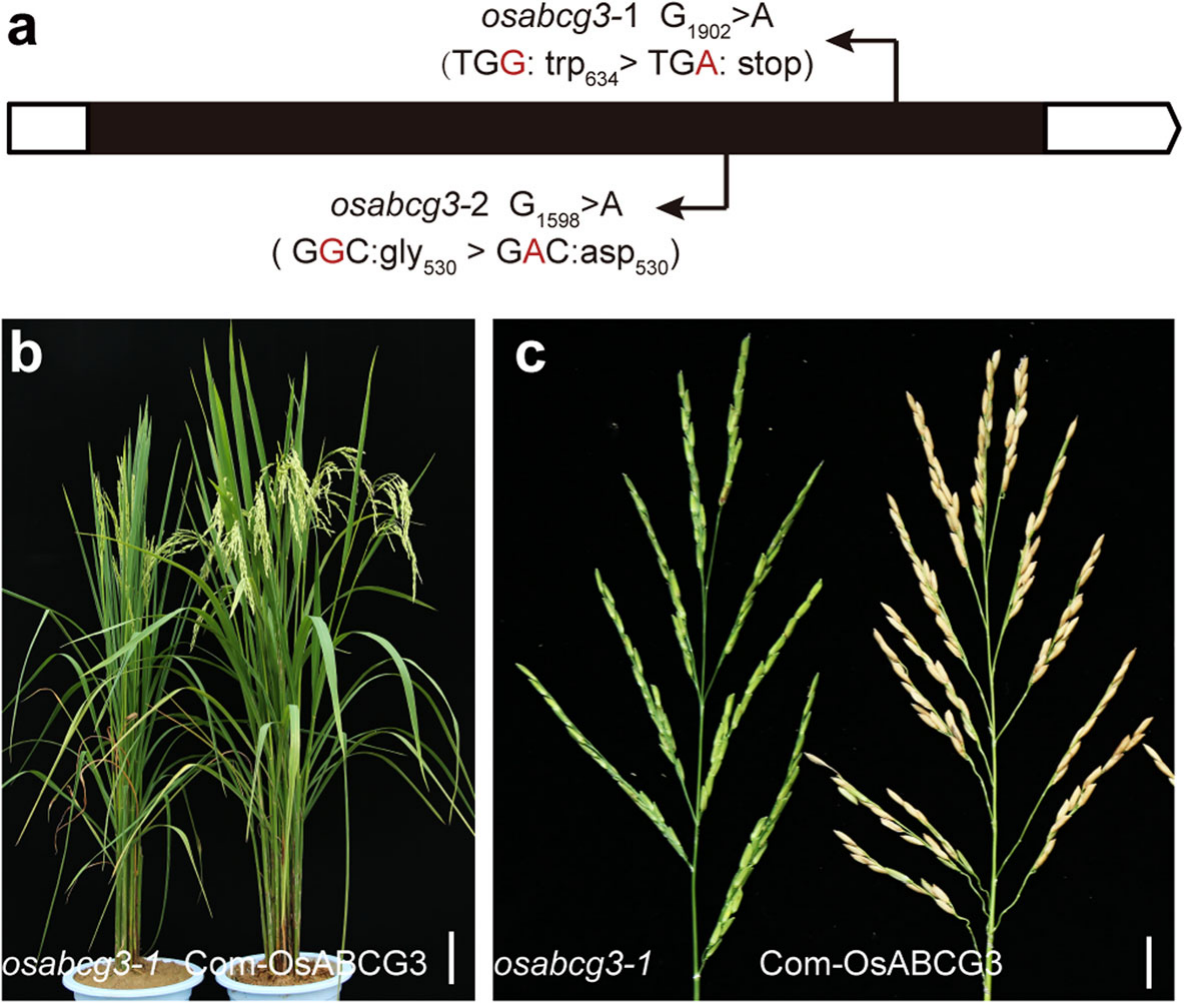
Identification and complementation of *OsABCG3*. **a** The gene structure of OsABCG3. Mutations of OsABCG3 in *osabcg3*–1 and *osabcg3*–2 were showed. Black box indicates exon, and white box indicates UTRs. **b, c** Transgenic complementation of the *osabcg3*–1 mutant. Plants (**b**) and seed set (**c**) of *osabcg3*–1 and transgenic *osabcg3*–1 mutant complemented with the *OsABCG3* fragment were showed. Scale bars = 10 cm (**b**) and 1 cm (**c**)

Sterile individuals in each F2 population mentioned above were bulk-sequenced. The sequence data were subjected to the Simultaneous Identification of Multiple Causal Mutations (SIMM) pipeline (Yan et al. 2017) for identification of the mutant gene. The analyses identified two different G > A mutations, one in each mutant, that were all located on *LOC_Os01g61940* (Fig. 2a; Additional file 2: Figure S2; Additional file 3: Table S1). *LOC_Os01g61940* encodes an ABC transporter named OsABCG3 that carries an ATPase-associated domain (residues 140–332) and six transmembrane domains (residues 463–743). The mutation in *osabcg3*–1 (G_1902_ > A) resulted in premature termination after the 633 residue, while in *osabcg3*–2 (G_1589_ > A) caused amino acid substitutions of G_530_D. All these mutation sites were near the transmembrane regions predicted by TMHMM Server v. 2.0 (http://www.cbs.dtu.dk/services/TMHMM/).

To verify the associations between mutations and sterility, we analyzed the genotype of F2 individuals using high resolution melting (HRM) analysis. All the male sterile plants carried the corresponding homozygous mutations, whereas the fertile plants showed 2:1 ratio of heterozygous and homozygous WT genotypes, suggesting *OsABCG3* being the causal gene of *osabcg3*–1 and *osabcg3*–2. Genetic complementation was performed to test the allelic relationship between these two mutants. By pollinating homozygous *osabcg3*–1 with pollen from heterozygous *osabcg3*–2, the plants combining mutations G_1902_ > A and G_1589_ > A were isolated from the progeny. These plants were completely male sterile (Additional file 4: Figure S3), indicating that *osabcg3*–1 and *osabcg3*–2 were allelic.

Additional alleles were generated using the CRISPR technology. The mutant lines carrying indel mutations, which led to frame shift and premature termination of *OsABCG3*, all exhibited defective pollen similar to *osabcg3*–1 and *osabcg3*–2 (Additional file 5: Figure S4). To further confirm the role of *OsABCG3,* the 6.7 kb genomic fragment for *OsABCG3*, including 2.5 kb upstream region, 2.3 kb coding region, and 1.9 kb downstream region, was introduced into homozygous *osabcg3*–1. Positive transgenic plants displayed normal seed set (Fig. 2b, c). Together, we concluded that mutations in *OsABCG3* caused the male sterility.

### Phylogenic analysis of OsABCG3

OsABCG3 belongs to the half-size ABC transporter of the G family, with 28 and 32 members in Arabidopsis and rice, respectively (Verrier et al. 2008). Seven known ABC transporters required for pollen wall formation, including AtABCG11 (Panikashvili et al. 2010), AtABCG26 (Quilichini et al. 2010; Quilichini et al. 2014), AtABCG9 (Choi et al. 2014), AtABCG1 and AtABCG16 (Yadav et al. 2014) in Arabidopsis, as well as OsABCG26 (Zhao et al. 2015; Chang et al. 2016b) and OsABCG15 (Niu et al. 2013a; Qin et al. 2013; Zhu et al. 2013; Wu et al. 2014) in rice, all belong to this family (Additional file 6: Figure S5). These ABCG transporters were proposed to allocate various lipidic, phenolic, and other sporopollenin precursors and tryphine components from the tapetum where they are generated to the anther locule for pollen wall formation (Zhao et al. 2016). Phylogenetic analysis revealed that, among the seven members, AtABCG1 and AtABCG16, which are required for the development of nexine and intine layers (Yim et al. 2016), are most closely related to OsABCG3. Other members in the OsABCG3 clan include AtABCG2, AtABCG6 and AtABCG20 that are required for suberin formation in Arabidopsis roots and seed coats (Yadav et al. 2014), and OsABCG5 that is required for suberization of rice root hypodermis (Shiono et al. 2014). These ABC transporters contribute to formation of extracellular barriers probably by transporting precursors for the hydrophobic polymers (Do et al. 2017).

### *OsABCG3* is mainly expressed in anther

**Fig. 3:**
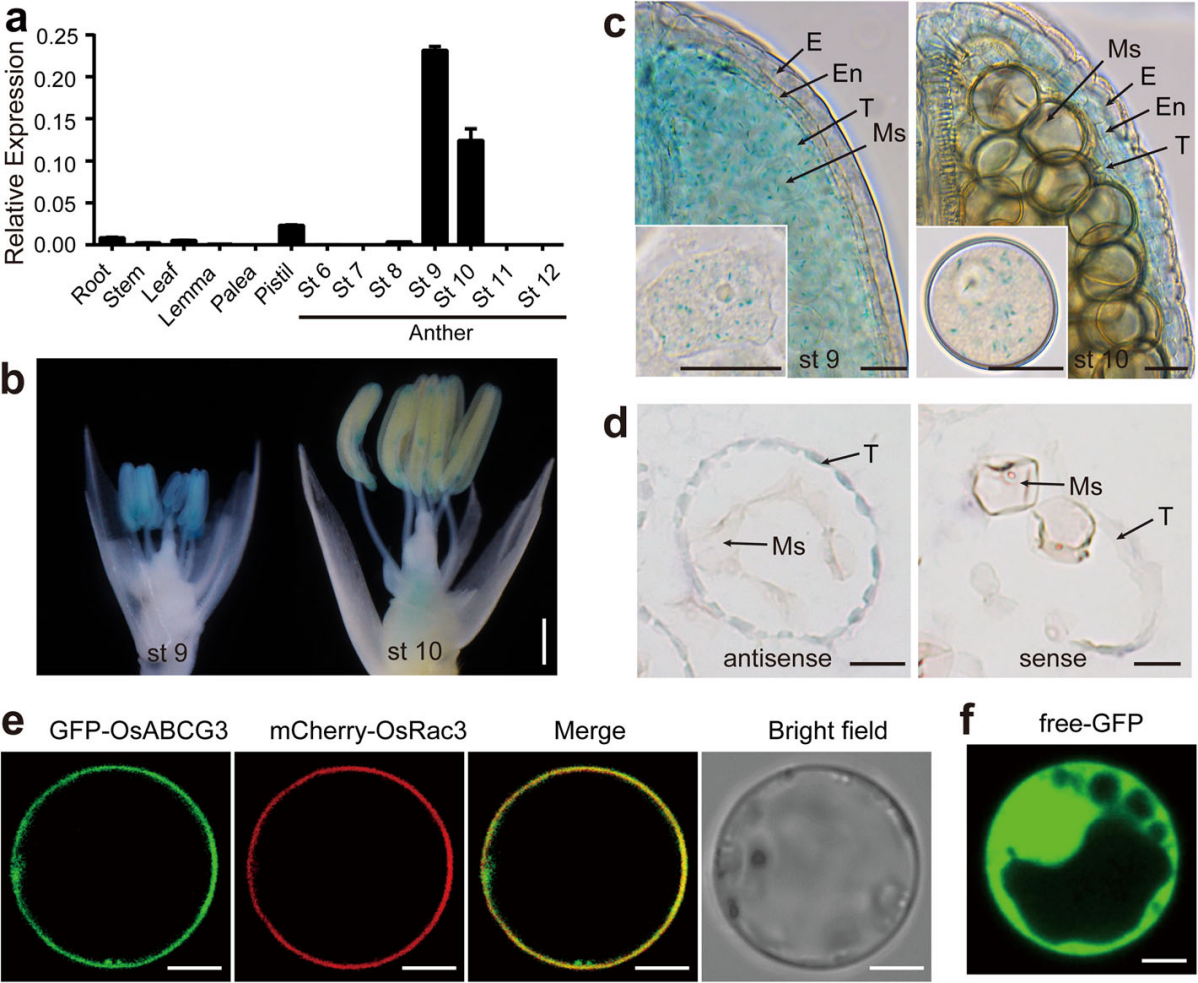
Expression pattern analysis of *OsABCG3* and subcellular localization analysis of OsABCG3 protein. **a** Expression pattern of OsABCG3. Anthers were collected at different developmental stages (St 6–12). Pistils and other tissues were harvested from plants at heading stage. The expression levels were determined by qRT-PCR. *OsACTIN1* was used as internal standards. Data are shown as means ± *SD* (*n* = 3). **b-c** GUS staining of the OsABCG3_pro_: GUS transgenic plant. The spikelets with the palea and lemma removed (**b**), and cleared mounted anther and microspores (**c**) were showed respectively. **d** In situ hybridization of *OsABCG3* antisense and sense probes with the WT anther sections, respectively. **e, f** Subcellular localization of OsABCG3-GFP fusion protein. The fusion protein GFP-OsABCG3 was co-expressed with mCherry-OsRac3, a plasma membrane marker, by transient expression in rice protoplasts **(e)**. The construct 35S:GFP was transformed as a control **(f)**. Localization of the fluorescence-tagged proteins was examined by confocal microscopy. Scale bars =1 mm (**b**); 25 μm (**c, d**); 5 μm (**e, f**)

All the *osabcg3* mutants exhibited abnormal anther development, whereas no obvious phenotype for vegetative growth was observed. To understand the dedicated role of *OsABCG3*, we analyzed the tissue specificity and developmental expression pattern of *OsABCG3* using quantitative reverse transcription-PCR (qRT-PCR). The results revealed that the *OsABCG3* expression was negligible in root, stem and leaf at heading stage, but relatively high in anther and pistil, particularly in anthers at developmental stages 9 to 10 when the formation of pollen exine began (Fig. 3a). To determine more precisely the spatial expression of *OsABCG3*, we introduced the construct containing the *OsABCG3* promoter fused with β-glucuronidase (GUS) into WT plants. GUS activity was apparent in anther at developmental stage 9 and 10, and relatively weak in the pistil-receptacle junction (Fig. 3b), which was consistent with qRT-PCR data. To obtain more details about the gene expression in anther, the stained anthers were observed by microscopy after cleared and mounted. The GUS signal was observed both in the tapetum and microspores, and the signal was stronger in the anther at stage 9 than stage 10 (Fig. 3c), which was consistent with the qRT-PCR result. To verify the result, we perform in situ hybridization of *OsABCG3* using the WT anther. A relatively strong hybridization signal was observed in the tapetum, but the signal was weaker in microspores (Fig. 3d), which was consistent with the GUS signal at stage 10. Despite the apparent expression in pistil, *osabcg3* mutants showed normal seed setting after manually pollinated with WT pollen (Additional file 1: Figure S1), suggesting that *OsABCG3* is not essential for female fertility.

The subcellular localization of OsABCG3 was determined by transient expression of GFP-OsABCG3 fusion protein in rice protoplasts. While the free GFP signal was visible in the whole cell, the fusion protein GFP-OsABCG3 was detected only on the plasma membrane, co-localized with the plasma membrane marker mCherry-OsRac3 (Fig. 3e, f). The plasma membrane localization of OsABCG3 is similar to the known anther development-associated ABC transporters, such as OsABCG15 and OsABCG26 (Zhao et al. 2015), implying that OsABCG3 is probably also involved in intercelluar transport.

### *OsABCG3* is required for pollen wall formation

**Fig. 4:**
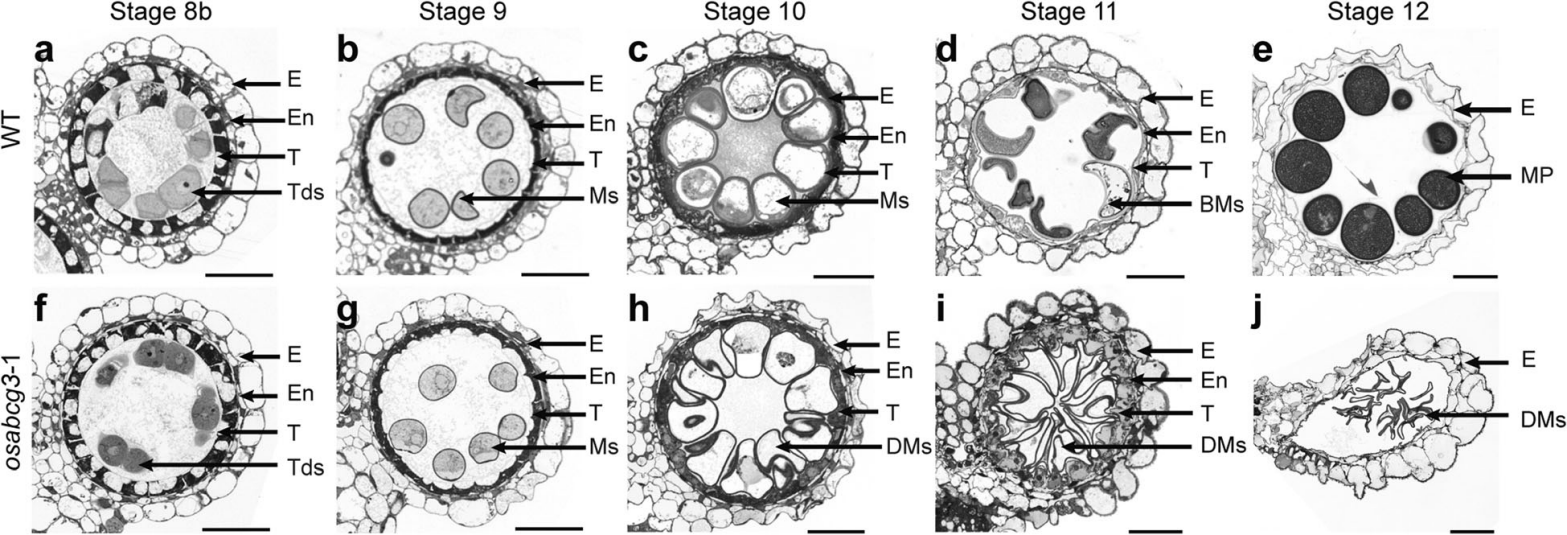
Transverse sections of anther in WT and *osabcg3*–1 from stage 8b to 12. WT anthers are showed in **a-e**. *osabcg3*–1 anthers are showed in **f-j**. BMs, binuclear microspores; DMs, degenerated microspores; E, epidermis; En, endothecium; MP, mature pollen; Ms., microspores; T, tapetal layer; Tds, tetrads. Scale bars = 20 μm

To understand how *OsABCG3* affects anther development, we analyzed the sections of *osabcg3* mutant anthers. In rice, anther development is divided into 14 stages based on morphological features (Zhang et al. 2011). Transverse sections indicated that, prior to stage 9, the microspores and anther wall of *osabcg3* was indistinguishable from the WT (Fig. 4a, b, f, g). At stage 10, the WT microspores were round and vacuolated, with the big vacuole pushing the cytoplasm to the cell periphery (Fig. 4c). However, the *osabcg3* mutant microspores were misshapen, and the cytoplasm appeared to shrink in the center (Fig. 4h). At the beginning of stage 11, the WT microspore further underwent first mitosis and became falcate, while the WT tapetum was almost completely degenerated (Fig. 4d). On the contrary, in *osabcg3*, microspore began to collapse, and the tapetum was swollen, showing abnormal tapetal degeneration (Fig. 4i). When the WT microspore developed into spherical pollen grains filled with cellular contents by stage 12, the mutant microspores aborted, leaving a shriveled cell wall eventually (Fig. 4e, j, Additional file 7: Figure S6a).

**Fig. 5:**
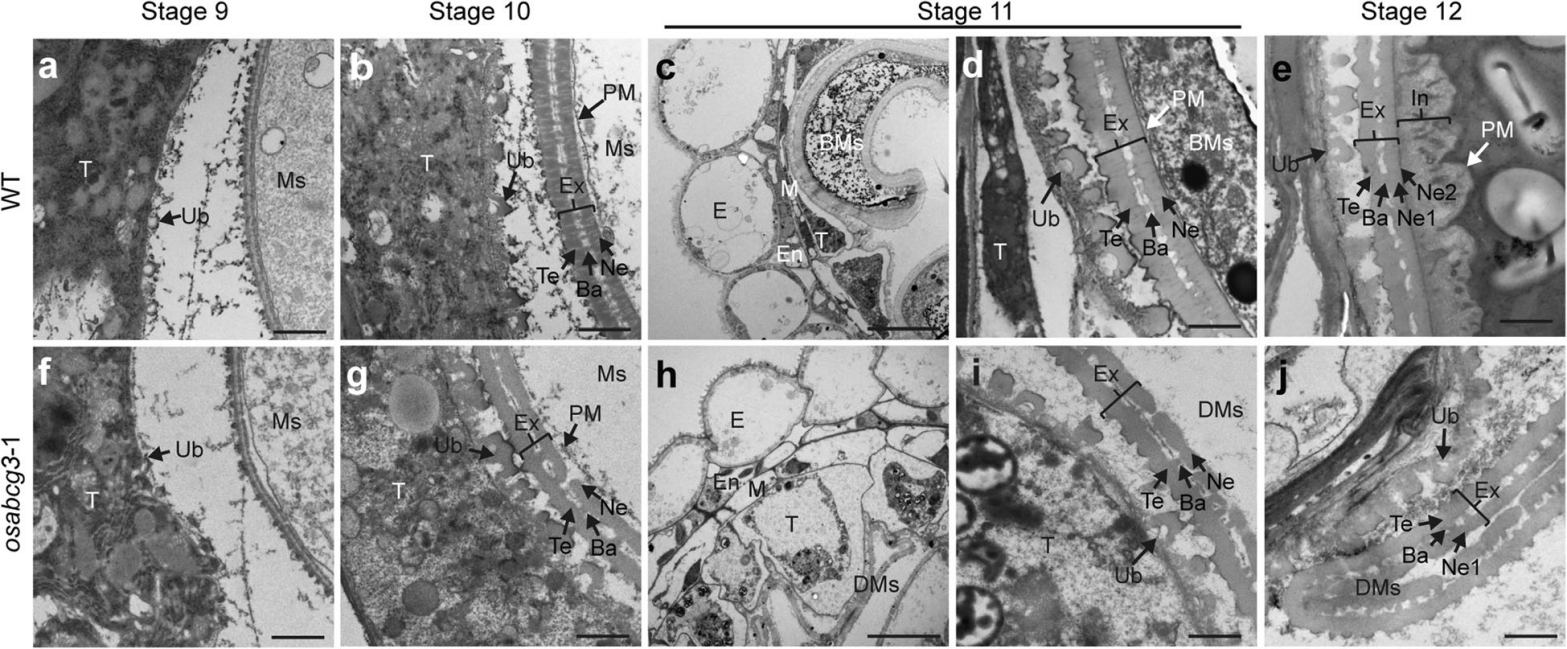
TEM analysis of the anther sections in WT and *osabcg3–*1 from stages 9 to 12. WT anthers are showed in **a-e,** and *osabcg3*–1 anthers are showed in **f-j**. Ba, bacula; BMs, binuclear microspores; DMs, degenerated microspore; E, epidermis; En, endothecium; Ex, exine; In, intine; M, middle layer; Ms., microspores; Ne, nexine; Ne1, nexine I; Ne2, nexine II; PM, plasma membrane; T, tapetal layer; Te, tectum; Ub, Ubisch body. Scale bars = 1 μm (**a-b**, **d-g**, **i**, **j**); 10 μm (**c, h**)

Electron microscopy was performed to obtain more details of the cellular defects. Tapetum is the main tissue providing signals and materials for microspore development (Scott et al. 2004). The WT tapetum underwent programmed cell death (PCD) from stage 8b, became highly condensed afterwards, and almost completely degenerated into cellular debris by stage 11 (Fig. 5a-d). The mutant tapetum did not show a clear difference from the WT at stage 9 (Fig. 5f). At stage 10, however, the mutant tapetum looked obviously different with an increased number of vacuoles and vesicles (Fig. 5g). Unlike the WT tapetum that almost completely degenerated by stage 11, the mutant tapetum further swelled and lacked distinct organelle structures (Fig. 5h, i), suggesting abnormal degradation of the tapetum.

In WT anther, the Ubisch bodies, which are thought to export sporopollenin precursors from the tapetum to the microspore, were generated at stage 9, and proexine was formed on the microspore simultaneously (Fig. 5a). At stage 10 and 11, the Ubisch bodies were more visible, showing transparent conical protrusions covered by dark-stained materials. In parallel, the two-layer exine surrounding the microspore was properly formed with a well-organized outer sexine (contained tectum and bacula) and the inner nexine (Fig. 5b, d). By stage 12, the pollen wall further thickened, and distinct structures of tectum, bacula, nexine I, nexine II, and intine were visible outside of the pollen plasma membrane (Fig. 5e).

In *osabcg3*, no clear difference was observed in Ubisch bodies and microspores by stage 9 (Fig. 5f). In stage 10, the Ubisch bodies seemed normal, and layers of tectum, bacula, and nexine were visible. However, the exine was less condensed, the nexine layer was discontinuous, and the microspore plasma membrane was fragmental and showing signs of cell degeneration (Fig. 5g). By stage 11 and 12, the Ubisch bodies of mutant were slightly smaller and less organized than that of the WT (Fig. 5d, i; Additional file 7: Figure S6a). Moreover, the plasma membrane and cellular contents were completely degenerated from the microspores, the pollen wall was thinner without the intine and nexine II layers (Fig. 5i, j), and pollen grains collapsed, and the framework of the exine distorted and folded (Fig. 5j; Additional file 7: Figure S6a). On the other hand, compared to WT, the cuticle on the anther epidermis seemed normal in *osabcg3* (Additional file 7: Figure S6a, b).

Together, these results indicated that loss-function of *OsABCG3* affected the tapetum degeneration and pollen wall formation. The cellular defects in *osabcg3* were different from what in *osabcg15* and *osabcg26* mutants that lack Ubisch bodies, pollen wall, and anther cuticle (Niu et al. 2013a; Qin et al. 2013; Zhu et al. 2013; Wu et al. 2014; Zhao et al. 2015; Chang et al. 2016b).

### Mutation in *OsABCG3* altered the transcript levels of the lipid transport genes

**Fig. 6:**
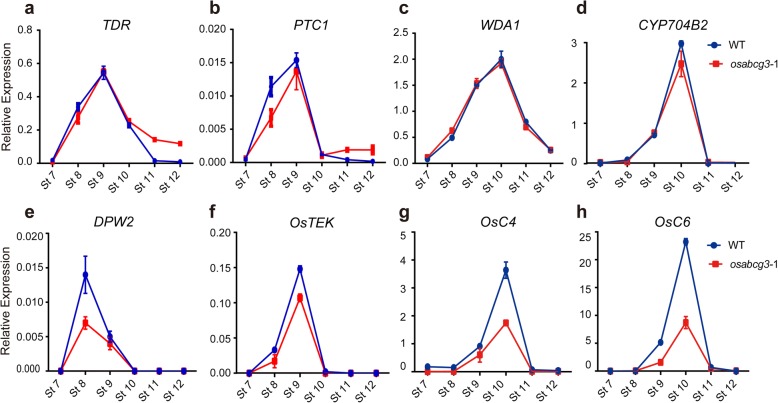
Expression of genes related to pollen development in the WT and *osabcg3*–1 anthers. Expression of *TDR* **(a)**, *PTC1* **(b)**, *WDA1* **(c)**, *CYP704B2* **(d)**, *DPW2* (**e**), *OsTEK* (**f**), *OsC4* **(g)** and *OsC6* **(h)** at stage 7 to 12 in the WT and *osabcg3*–1 was analyzed using qRT-PCR. *OsACTIN1* served as an internal control. Data are shown as means ± *SD* (*n* = 3)

**Fig. 7:**
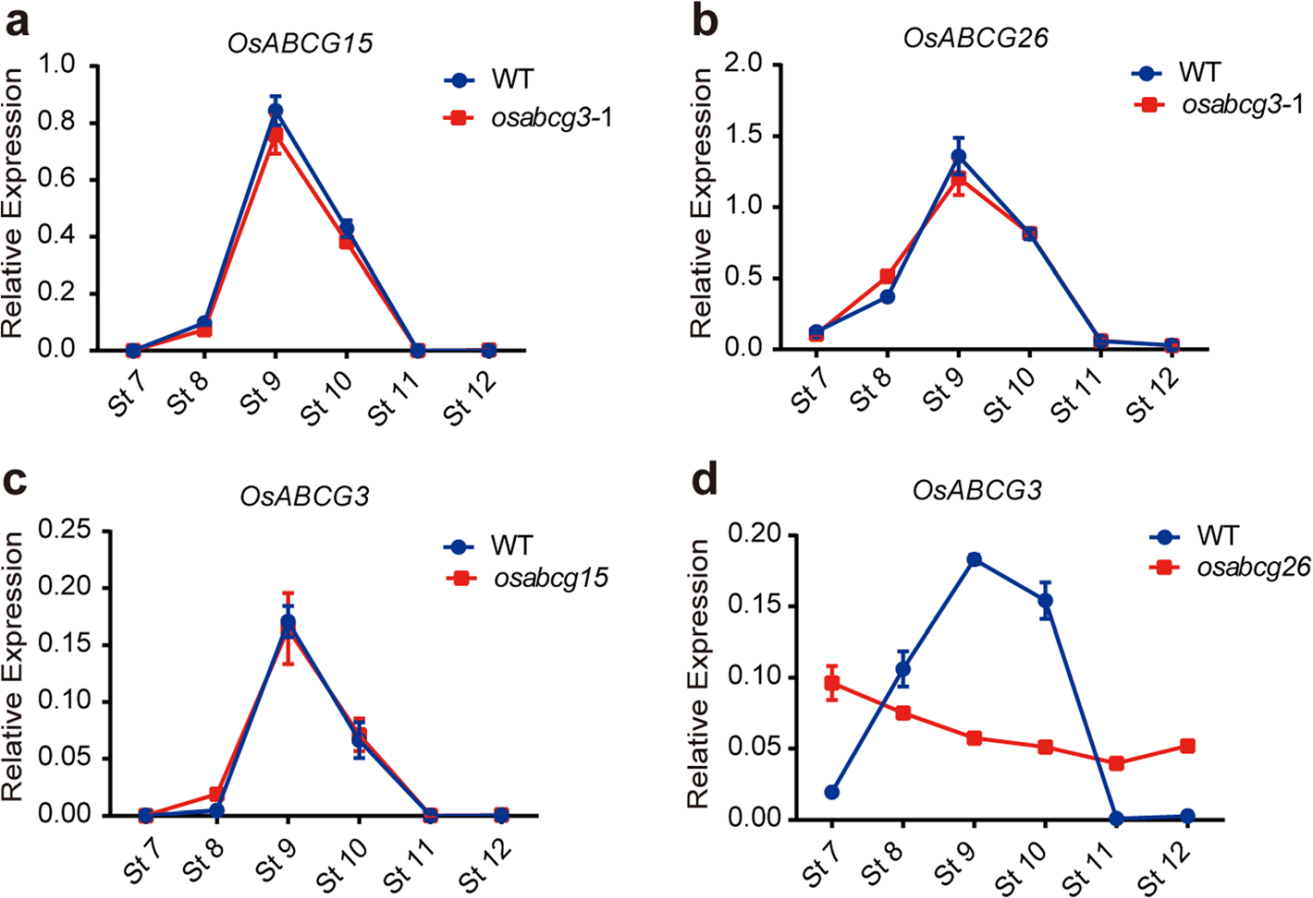
Expression of OsABCGs in WT and mutant anthers. Expression of *OsABCG15* in the WT and *osabcg3*–1 **(a)**, *OsABCG26* in the WT and *osabcg3*–1 **(b)**, *OsABCG3* in the WT and *osabcg15* **(c)**, and *OsABCG3* in the WT and *osabcg26* **(d)** were analyzed using qRT-PCR. *OsACTIN1* served as an internal control. Data are shown as means ± *SD* (*n* = 3)

Pollen wall formation involves complicate gene expression networks, including transcription factors, sporopollenin synthesizing enzymes, and transporters, that are tightly regulated in the tapetum (Shi et al. 2015). To understand the role of *OsABCG3* in tapetum development and pollen development, we compared the expression patterns of a few key genes essential for tapetum degeneration and pollen wall formation between the WT and *osabcg3* by qRT-PCR analysis. Mutation of *OsABCG3* did not alter the expression of the regulatory genes *TDR* (Zhang et al. 2008) and *PERSISTANT TAPETAL CELL 1* (*PTC1*) (Li et al. 2011), and the aliphatic lipid metabolism genes *WAX-DEFICIENT ANTHER 1* (*WDA1*) and *CYP704B2* (Jung et al. 2006; Li et al. 2010a). However, the expression levels of *DPW2* in aromatic lipids/phenolics biosynthesis pathway (Xu et al. 2017), *TEK* necessary for nexine formation (Lou et al. 2014; Jia et al. 2015) and lipid transport genes such as *OsC4* and *OsC6* (Zhang et al. 2010)*,* were reduced in mutant anther (Fig. 6). However, in *osabcg15* and *osabcg26* mutants, the expression of *TDR*, *WDA1*, *CYP704B2, OsC4* and *OsC6* was all reduced, but the *PTC1* transcript was increased (Niu et al. 2013a; Qin et al. 2013; Zhu et al. 2013; Wu et al. 2014; Zhao et al. 2015; Chang et al. 2016b). These results suggested that *OsABCG3* regulates lipid metabolisms and transport during anther development in a way that different from *OsABCG15* and *OsABCG26.* To determine if *OsABCG3, OsABCG15* and *OsABCG26* are transcriptionally related*,* we investigated how mutation of one gene impacted the expression of the other two genes. As shown in Fig. 7, mutation of *OsABCG3* did not change the expression of *OsABCG15* and *OsABCG26* (Fig. 7a, b), nor did mutation of *OsABCG15* on the expression of *OsABCG3* (Fig. 7c). But mutation of *OsABCG26* altered the expression kinetics of *OsABCG3* (Fig. 7d). These results suggested that OsABCG3, OsABCG15, and OsABCG26 regulate pollen development through discrete yet interacting pathways.

## Discussion

The isolation and characterization of rice male sterile mutants allowed us to identify two allelic *osabcg3* mutants that were defective in pollen development. OsABCG3 belongs to the half-size ABCG transporter family. Many members in this family are known to be involved in the formation of hydrophobic polymers such as cutin, suberin, pollen coat and sporopollenin, presumably by allocating various lipidic and phenolic compounds from the site they are produced to the place where they are polymerized (Zhao et al. 2016; Do et al. 2017). Given that the fusion protein GFP-OsABCG3 was localized on the plasma membrane and loss-function of OsABCG3 caused sporophytic male sterility related to defective pollen, we speculated that OsABCG3 probably played a role in transporting materials from the tapetum to the microspores.

In addition to OsABCG3, two other members in the half-size ABCG family, OsABCG15 and OsABCG26, are also required for pollen development and male fertility in rice (Zhang et al. 2011; Niu et al. 2013a; Qin et al. 2013; Zhu et al. 2013; Wu et al. 2014; Zhao et al. 2015; Chang et al. 2016b). *OsABCG3*, *OsABCG15* and *OsABCG26* are all preferentially expressed in anthers, and the three genes show similar expression kinetics during anther development by starting the expression at the tetrad stage (stage 8) and peaking when microspores are released from tetrad (stage 9) (Zhang et al. 2011; Niu et al. 2013a; Qin et al. 2013; Zhu et al. 2013; Wu et al. 2014; Zhao et al. 2015; Chang et al. 2016b). Although loss-function of either gene causes complete male sterility, our microscopic observations indicated that *OsABCG3* likely regulates pollen development in a way that is different from *OsABCG15* and *OsABCG26*. The *osabcg15* and *osabcg26* mutants do not have pollen exine, which leads to rupture of microspores before first mitosis, and the mutant plants exhibit a “no pollen” phenotype (Zhang et al. 2011; Niu et al. 2013a; Qin et al. 2013; Zhu et al. 2013; Wu et al. 2014; Zhao et al. 2015; Chang et al. 2016b). Mutations on *OsABCG15* and *OsABCG26* also abolish the anther surface cuticle development (Zhang et al. 2011; Niu et al. 2013a; Qin et al. 2013; Zhu et al. 2013; Wu et al. 2014; Zhao et al. 2015; Chang et al. 2016b). Based on these observations, Zhao et al. (2015) proposed that OsABCG15 and OsABCG26 act as partners to allocate aliphatic and aromatic precursors from the tapetum for pollen wall and anther surface cuticle formation. Different from *osabcg15* and *osabcg26* mutants, *osabcg3* mutant displayed a well preserved pollen exine framework, and the anther cuticle looked normal in *osabcg3* mutant. These differences suggest that OsABCG3 and OsABCG15 and OsABCG26 likely transport different materials from the tapetum for microspore development.

The functional difference of *OsABCG3* from *OsABCG15* and *OsABCG26* was also reflected by the interactions at the gene expression level. Mutation of *OsABCG3* did not affect the expression of *OsABCG15* and *OsABCG26*, and neither mutation of *OsABCG15* affected the expression of *OsABCG3*, suggesting that *OsABCG3* and *OsABCG15* act in independent pathways. However, mutation of *OsABCG26* altered the expression kinetics of *OsABCG3*, suggesting a possible epistatic relationship between *OsABCG26* and *OsABCG3* presumably involving the OsABCG26 substrates in a feedback regulation. These observations also raised a possibility that OsABCG15 and OsABCG26 may transport different substrates from the tapetum rather than work as partners, although their corresponding mutants showed the similar morphological phenotype.

In addition to functional differences for pollen development, the three genes also displayed distinct expression patterns in the female reproductive organ. *OsABCG15* is not expressed in pistil, but *OsABCG26* and *OsABCG3* showed substantial expression in pistil (Zhang et al. 2011; Niu et al. 2013a; Qin et al. 2013; Zhu et al. 2013; Wu et al. 2014; Zhao et al. 2015; Chang et al. 2016b). *OsABCG26* is mainly expressed in stigma and the top part of the ovary, and it is required for pollen tube growth in the transmitting track (Chang et al. 2016b). *OsABCG3* is specifically expressed in the pistil-receptacle junction, and our pollination assay indicated that OsABCG3 is dispensable for female fertility.

The tapetum PCD is critically important to the pollen development. At the time when meiosis begins, the tapetum cells initiate the PCD by showing cytoplasm condensation and signals of genomic DNA fragmentation (Zhang et al. 2011). Further development of the microspores is accompanied by further progression of tapetal PCD, which is associated with more condensed and vacuolated cytoplasm, then the formation of Ubisch bodies, and eventual degeneration of the tapetal cells (Zhang et al. 2011). Cytological analysis revealed that the tapetal PCD was normal in *osabcg3* at stage 9 when microspores were just released from the tetrads, but soon after signs of abnormality occurred to the *osabcg3* tapetum. The mutant tapetum was lightly stained at stage 10 and enlarged afterwards, indicating abnormal degradation. Compared to the other mutants of tapetum development regulators, such as *GAMYB*, *TDR, ETERNAL TAPETUM 1* (*EAT1*) and *PTC1* (Zhang et al. 2008; Aya et al. 2009; Li et al. 2011; Niu et al. 2013b), the tapetum defects in *osabcg3* appeared later and minor. Moreover, our qRT-PCR analysis showed that at the developmental stage when *OsABCG3* was expressed, the expression levels of *TDR* and *PTC1* in *osabcg3* anther were parallel to the WT plants. These results suggested that mutation of OsABCG3 affects tapetum development via a specific pathway rather than a feedback regulation on transcriptional cascades operated by the known transcriptional regulators.

A major defect exhibited by the *osabcg3* pollens was on the pollen wall. The pollen exine in *osabcg3* was visible with the sexine and discontinuous nexine I, but nexine II and the intine were disappeared. It has long been assumed that pollen exine development is contributed by tapetum, while intine synthesis is largely under the control of microspores (Shi et al. 2015). In fact, a number of genes required for the intine development, such as *GLYCOSYL TRANSFERASE 1* (*GT1*) and *COLLAPSED ABNORMAL POLLEN 1* (*CAP1*) in rice, *UDP-SUGAR PYROPHOSPHORYLASE* (*AtUSP*) and *FASCICLIN-LIKE ARABINOGALACTAN-PROTEINS 3* (*AtFLA3*) in Arabidopsis, regulate pollen fertility in a gametophytic manner (Li et al. 2010b; Geserick and Tenhaken 2013; Moon et al. 2013; Ueda et al. 2013). *OsABCG3* is expressed in both the tapetum and microspores. However, given that the pollen phenotype in *osabcg3* mutant was inherited sporophytically, we proposed that the *OsABCG3* expressed by the tapetum was mainly responsible for the pollen wall defect. The *OsABCG3* expressed by the microspores did not play an important role, because the pollen grains produced by the *osabcg3* heterozygous plant were all normal. How does the tapetum-produced ABC transporter regulate the intine formation? One possibility is that the intine formation requires not only the gametophyte-produced materials but also the tapetum-produced materials, and OsABCG3 is involved in the translocation of the tapetum-produced materials to the microspores. Another possibility is that the OsABCG3-transported compound(s), which may serve as nutrients, are necessary to nurture the microspore cytoplasm, and lack of the compounds leads to degeneration of the microspore cytoplasm. It is known in rice that microspores initiates the intine formation by the end of the bicellular pollen stage (stage 11) when exine development is almost finished (Zhang et al. 2011; Shi et al. 2015; Zhang et al. 2016). However, we found that microspores of *osabcg3* mutant showed plasma membrane breakage by stage 10 and complete cytoplasm degeneration by stage 11, long before the intine formation. Thus the lack of intine is expected, because degraded microspore cytoplasm cannot synthesize the intine. The third possibility is that the OsABCG3-allocated material(s) is essential for proper construction of the exine, and a fully functional exine is required for the intine formation. This speculation is supported by recent investigations on the Arabidopsis *tek* mutant and rice *dpw2* mutant that both have the exine framework but lack the intine (Lou et al. 2014; Xu et al. 2017). TEK is a AHL family transcriptional regulator regulating a group of arabinogalactan proteins necessary for nexine formation (Lou et al. 2014; Jia et al. 2015), while DPW2 is a putative BAHD acyltransferase capable of transferring hydroxycinnamic acid moieties in vitro, using ω-hydroxy fatty acids as acyl acceptors and hydroxycinnamoyl-CoAs as acyl donors (Xu et al. 2017). Both *TEK* and *DPW2* are sporophytic genes and are preferentially expressed in the tapetum (Lou et al. 2014; Xu et al. 2017). The *tek* mutant does not have the nexine and intine layer (Lou et al. 2014), while the *dpw2* mutant does not have the nexine II and intine (Xu et al. 2017). In both cases, the absence of the nexine layer was proposed to be accountable for the lack of the intine formation (Lou et al. 2014). The *osabcg3* mutant pollen did not have the nexine II layer. Therefore, the absence of intine in *osabcg3* may have arisen from the lack of nexine II. It is possible that the nexine protects the microspore cytoplasm from collapse after they are released from the tetrad until the time the intine is formed.

Previous studies have shown that rice sporopollenin and anther cuticle share common precursors that are synthesized in the tapetum (Shi et al. 2015; Zhang et al. 2016). Both aliphatic and aromatic lipids are important components of pollen exine and anther cuticle (Ariizumi and Toriyama 2011; Shi et al. 2015). Genes related to aliphatic lipids metabolisms contribute to the development of exine and anther cuticle profoundly. For instance, null mutants of rice genes *OsCYP704B2*, *OsCYP703A3*, *DPW*, *WDA1,* and *OsACOS12* have flat cuticle and lack Ubisch bodies and pollen exine (Jung et al. 2006; Li et al. 2010a; Shi et al. 2011; Yang et al. 2014; Li et al. 2016; Yang et al. 2017). Interestingly, the *OsABCG15* and *OsABCG26* mutants display the same phenotype as mutants of the aliphatic lipids metabolism genes (Niu et al. 2013a; Qin et al. 2013; Zhu et al. 2013; Wu et al. 2014; Zhao et al. 2015; Chang et al. 2016b) suggesting a possible role of OsABCG15 and OsABCG26 in allocation of aliphatic lipids. On the other hands, mutants of genes related to aromatic lipids biosynthesis, such as *PKSA/B* (Kim et al. 2010; Dobritsa et al. 2010), *TKPR1/2* (Grienenberger et al. 2010), *OsPKS1/2* (Wang et al. 2013; Zhu et al. 2017; Zou et al. 2018) and *DPW2* (Xu et al. 2017), have relatively minor effects on anther cuticle and pollen exine. In *osabcg3* mutant, the development of anther cuticle was normal, and the exine skeleton looked similar to that of the wild type except the exine was less condensed and the nexine II is absent. These cytological characteristics were similar to what in *ospks2* and *dpw2* (Xu et al. 2017; Zhu et al. 2017; Zou et al. 2018). Different from *OsABCG15* and *OsABCG26,* mutation of *OsABCG3* caused reduction of transcription level of *DPW2*, rather than *WDA1* and *CYP704B2*. Therefore, it is possible that *OsABCG3* is involved in transporting aromatic lipids rather than aliphatic lipids.

OsABCG3 was more closely related to AtABCG1 and AtABCG16 than to OsABCG15 and OsABCG26 in protein sequence. The pollen wall defects in *osabcg3* were also similar to those in the *atabcg1 atabcg16* double mutant (Yadav et al. 2014). AtABCG1 and AtABCG16 were proposed to transfer precursors from the tapetum for nexine and intine formation, but the chemical nature of their substrates remains unknown (Yim et al. 2016). It is worthy to notice that AtABCG16 is reported to be involved in abscisic acid (ABA) and jasmonate tolerance (Ji et al. 2014), though the mechanism remains to be further clarified. In addition, several other ABC transporters contribute to phytohormone transport during anther development. For instance, AtABCG31 is essential for pollen coat formation and pollen viability under cold stress (Choi et al. 2014), and AtABCG31 was proposed to transfer ABA induced in the tapetum under cold stress (Baron et al. 2012). Given the similarity on protein sequences of OsABCG3 and AtABCG16, as well as the mutant phenotype, there is another possibility that OsABCG3 may contribute to pollen development via regulating hormone transport.

## Conclusions

In summary, this work elucidated the important role of *OsABCG3* in pollen development in rice. Ultrastructural analyses suggested that OsABCG3 and OsABCG15 and OsABCG26 likely transport different materials for pollen development. Because the chemical composition of pollen wall is complex, and the binding of ABCG transporters with their substrates is difficult to measure, we are still unable to determine the OsABCG3 substrates. Nonetheless, the identification and characterization of *osabcg3* mutant provided a new perspective to the understanding of molecular mechanisms governing the pollen wall development in rice.

## Methods

### Plant material and growth conditions

The *osabcg3*–1 and *osabcg3*–2 mutants were isolated from an Huanghuazhan (HHZ) mutant library generated by EMS treatment (Chen et al. 2014). *osabcg15* and *osabcg26* mutants were isolated from the same library and reported previously (Chang et al. 2016b). The F2 populations were generated from a cross of *osabcg3*–1 and *osabcg3*–2 with WT HHZ, and then selfed. The *osabcg3*–1/*osabcg3*–2 mutant genotypes were obtained by crossing *osabcg3*–1 homozygous with *osabcg3*–2 heterozygous plants. All plants were grown in the paddy field in Shenzhen.

### Characterization of mutant phenotype

Plants and flowers at mature stage were photographed with a Nikon D80 Digital Camera. For pollen fertility analysis, pollen grains at mature stage were stained with I_2_-KI solution and photographed using Nikon AZ100 microscope. To test the female fertility of *osabcg3*–1 and *osabcg3*–2, these mutants were cross-pollinated with the WT HHZ pollen manually. *osnp1–*1 male sterile mutant plants were used as a control (Chang et al. 2016a). For transverse section and electron microscopic analysis of anthers, spikelets at different developmental stages were collected and treated as described previously (Chang et al. 2016b).

### Molecular cloning of *OsABCG3*

The mutant genes of *osabcg3*–1 and *osabcg3*–2 were cloned with a SIMM method as described (Yan et al. 2017). Briefly, the *osabcg3*–1 and *osabcg3*–2 mutant plants were backcrossed with WT HHZ, and 30 sterile individuals in each F2 population were collected and bulk-sequenced respectively. The sequence data were subjected to computational analysis for identification of the mutant gene as described (Yan et al. 2017). Co-segregation of the candidate mutation with the phenotype in F2 population was analyzed using HRM analysis (Lochlainn et al. 2011). The primer sets HRM-osabcg3–1 and HRM-osabcg3–2 for HRM assay are listed in Additional file 8: Table S2.

### Plasmid construction and transformation

To create additional mutant alleles of *OsABCG3*, the CRISPR genome targeting system (Ma et al. 2015) was applied to generate construct CRISPR-OsABCG3, with target site (GGTGTTACTCCTCCTCCGCC) specifically for *OsABCG3.* The 6.7 kb HHZ genomic DNA fragment for *OsABCG3*, including the 2.5 kb upstream region, 2.3 kb coding region, and 1.9 kb downstream region, was PCR-amplified using primer set Com-OsABCG3-P1. The PCR products were cloned into the binary vector pCAMBIA1300 using InFusion HD cloning kit (Takara, Dalian, China) to generate construct Com*-*OsABCG3 for genetic complementation. For promoter analysis, the 2.5 kb upstream region of *OsABCG3* was PCR-amplified using the primer set Pro-OsABCG3-P1, and cloned into binary vector pHPG using InFusion HD cloning kit to yield OsABCG3_pro_:GUS. All constructs were sequence-confirmed before transformation. Constructs were introduced into *Agrobacterium tumerfaciens* AGL0 strain for rice transformation, as described previously (Chang et al. 2016b).

Com*-*OsABCG3 was introduced into the progeny of *osabcg3–*1 heterozygote plants. OsABCG3_pro_:GUS and CRISPR-OsABCG3 were introduced into *japonica* cv. Wuyungeng 7. The positive transgenic lines were determined by PCR, with primer set Pro-OsABCG3-P2 for OsABCG3_pro_:GUS construct, primer set SP-LR for CRISPR-OsABCG3, and primer set Com-OsABCG3-P2 for Com*-*OsABCG3. To identify the background genotype of Com*-*OsABCG3 transgenic plants, specific genomic fragment covering the mutation site of *osabcg3–*1 was amplified using primer set Com-OsABCG3-P3, and the product was further subjected to HRM analysis with primer set HRM-osabcg3–1. For transgenic lines of CRISPR-OsABCG3, DNA was amplified and sequenced using primer set CR-OsABCG3-Seq. All the primers are listed in Additional file 8: Table S2.

### Protein alignment and phylogenetic analysis

The protein sequences of 30 half-size ABCGs in rice and 28 half-size ABCGs in Arabidopsis (Verrier et al. 2008) were retrieved from RGAP (http://rice.plantbiology.msu.edu/) and TAIR (http://www.arabidopsis.org/) respectively. These proteins were aligned using ClustalW with default parameters, and the phylogenetic tree was constructed using the Neighbor-Joining algorithm (5,000 replicates) in MEGA7 (Kumar et al. 2016).

### Gene-expression analysis, histochemical GUS assay and in situ hybridization

Rice tissues were collected at the reproductive stage for qRT-PCR analysis of gene expression levels. Total RNA was extracted and then reverse-transcribed. qRT-PCR was performed with an Applied Biosystems 7500 Real-Time PCR System. Each experiment was biologically repeated three times, each with three replicates. *OsACTIN1* was used as the normalized reference. The relative expression levels were measured using the 2^−ΔCt^ analysis method. Primer sequences for *TDR*, *PTC1*, *WDA1*, *CYP704B2*, *DPW2*, *OsC4*, *OsC6*, *OsABCG15* and *OsABCG26* were designed according to the published genes (Jung et al. 2006; Zhang et al. 2008; Li et al. 2010a; Zhang et al. 2010; Li et al. 2011; Qin et al. 2013; Chang et al. 2016b; Xu et al. 2017). The TEK protein sequence in Arabidopsis (Lou et al. 2014; Jia et al. 2015) were used as the query sequence to blast in rice protein database and the most similar protein LOC_Os02g25020 were designated as OsTEK. All the primers are listed in Additional file 8: Table S2. Histochemical GUS stain was performed as described (Chang et al. 2016a) and photographed using Nikon AZ100 microscope. The stained anthers were then immersed into the clearing reagent, and then mounted into the slide and photographed using a Leica DM6 microscope, as described previously (Li et al. 2010a). In situ hybridization assay was performed as described (Chang et al. 2016a), using specific primer sets ISH-OsABCG3-S and ISH-OsABCG3-AS to generate sense and antisense probes respectively.

### Subcellular protein localization

To construct the GFP-OsABCG3 fusion protein, the open reading frame of *OsABCG3* was amplified from cDNA by PCR using primer set CDs-OsABCG3. The amplified cDNA fragment was then cloned in frame after the GFP coding region into the *pYL322-GFP* vector. To construct a plasma membrane-localized fluorescence marker protein mCherry-OsRac3 (Chen et al. 2010), the full-length coding sequence of *OsRac3* was amplified from the Nipponbare cDNA using primer set CDs-OsRac3. The resulting fragment was then cloned in frame after the mCherry coding region into the *p35S-mCherry-NosT* vector by the Ω-PCR strategy (Chen et al. 2013). The construct GFP-OsABCG3, mCherry-OsRac3 or control vector was introduced into rice leaf protoplasts by PEG-mediated transformation as previously described (Chen et al. 2011a). After transformation, cells were incubated at 30 °C dark for 12 to 15 h. The fluorescence signal was observed with a confocal laser scanning microscope (TCS SP8, Leica).

## Additional files

Additional file 1: **Figure S1.** Female fertility of *osabcg3*–1 and *osabcg3*–2 mutants. The seed set of *osnp1*–1, *osabcg3*–1 and *osabcg3*–2 after manually pollinated with WT pollen. Scale bars = 1 cm. (TIF 2498 kb)

Additional file 2: **Figure S2.** Determination of the candidate mutation sites for *osabcg3*–1 and *osabcg3*–2. Loess curve was used to show the distribution of ED^6^ values of all SNPs along the chromosomes. The candidate regions harboring causal mutation were indicated by red arrows on chromosome 1. (TIF 282 kb)

Additional file 3: **Table S1.** SIMM analyses of *osabcg3*–1 and *osabcg3*–2 mutants. (DOC 41 kb)

Additional file 4: **Figure S3.** Pollen grains of double heterozygote of *osabcg3*–1/*osabcg3*–2. Pollen grains of WT and *osabcg3*–1/*osabcg3*–2 double heterozygote were stained with I_2_-KI. Scale bars = 100 μm. (TIF 1208 kb)

Additional file 5: **Figure S4.** Characterization of *osabcg3* mutants in *janponica* Wuyungeng 7. **a** The site on *OsABCG3* targeted by the CRISPR/Cas9 system. The arrowhead indicates the target site. **b** *OsABCG3* sequence of three CRISPR-mutated lines in Wuyungeng 7 background*.* **c-e** Phenotype of WT and CRISPR-mutated plants. Plants at heading stage (**c**), spikelets with palea and lemma removed (**d**), and pollen grains stained with I_2_-KI (**e**) were showed. Scale bars = 10 cm (**c**); 1 mm (**d**); 100 μm (**e**). (TIF 1789 kb)

Additional file 6: **Figure S5.** Phylogenetic analysis of half-size ABCG proteins in rice and Arabidopsis. The half-size ABCG proteins in rice and Arabidopsis were aligned and used to construct a tree with MEGA7 software using the Neighbor-Joining method. OsABCG3 was marked in red, seven proteins required for pollen wall formation in blue, proteins for suberin formation in green, respectively. (TIF 562 kb)

Additional file 7: **Figure S6.** SEM and TEM analysis of the pollen grain and anther surfaces at stage 12 in WT and *osabcg3–*1. **a** SEM analysis of the pollen grain, Ubisch body and anther epidermis in WT and *osabcg3*–1 at stage 12. **b** TEM observation of the anther cuticle at stage 12. Scale bars = 5 μm (**a**); 500 nm (**b**). (TIF 2237 kb)

Additional file 8: **Table S2.** Primers used in this study. (DOC 56 kb)

## Abbreviations

ABA: Abscisic acid
ABC: ATP-binding cassette
*ACOS5*: *ACYL-COA SYNTHASE 5*
*AMS*: *ABORTED MICROSPORES*
*AtFLA3*: *FASCICLIN-LIKE ARABINOGALACTAN-PROTEINS 3*
*AtUSP*: Arabidopsis *UDP-SUGAR PYROPHOSPHORYLASE*
*CAP1*: *COLLAPSED ABNORMAL POLLEN 1*
*DPW*: *DEFECTIVE POLLEN WALL*
*DPW2*: *DEFECTIVE POLLEN WALL 2*
*DYT1*: *DYSFUNCTIONAL TAPETUM1*
*EAT1*: *ETERNAL TAPETUM 1*
EMS: Ethyl methanesulfonate
*GT1*: *GLYCOSYL TRANSFERASE 1*
GUS: β-glucuronidase
HHZ: Huanghuazhan
HRM: High resolution melting
*MS2*: *MALE STERILITY 2*
PCD: programmed cell death
*PKSA/B*: *POLYKETIDE SYNTHASE A/B*
*PTC1*: *PERSISTANT TAPETAL CELL 1*
qRT-PCR: Quantitative reverse transcription-PCR
SIMM: Simultaneous Identification of Multiple Causal Mutations
*TDF1*: *DEFECTIVE in TAPETAL DEVELOPMENT and FUNCTION 1*
*TDR*: *TAPETUM DEGENERATION RETARDATION*
*TEK*: *TRANSPOSABLE ELEMENT SILENCING* via *AT-HOOK*
*TKPR1/2*: *TETRAKETIDE TETRAKE REDUCTASE 1/2*
*WDA1*: *WAX-DEFICIENT ANTHER 1*
WT: Wild-type
