# Supplementary figures and images for: The ATP-binding cassette (ABC) transporter OsABCG3 is essential for pollen development in rice
Source: Rice (N Y). 2018 Oct 11;11:58. doi: 10.1186/s12284-018-0248-8 (PMC6181869; doi:10.1186/s12284-018-0248-8)

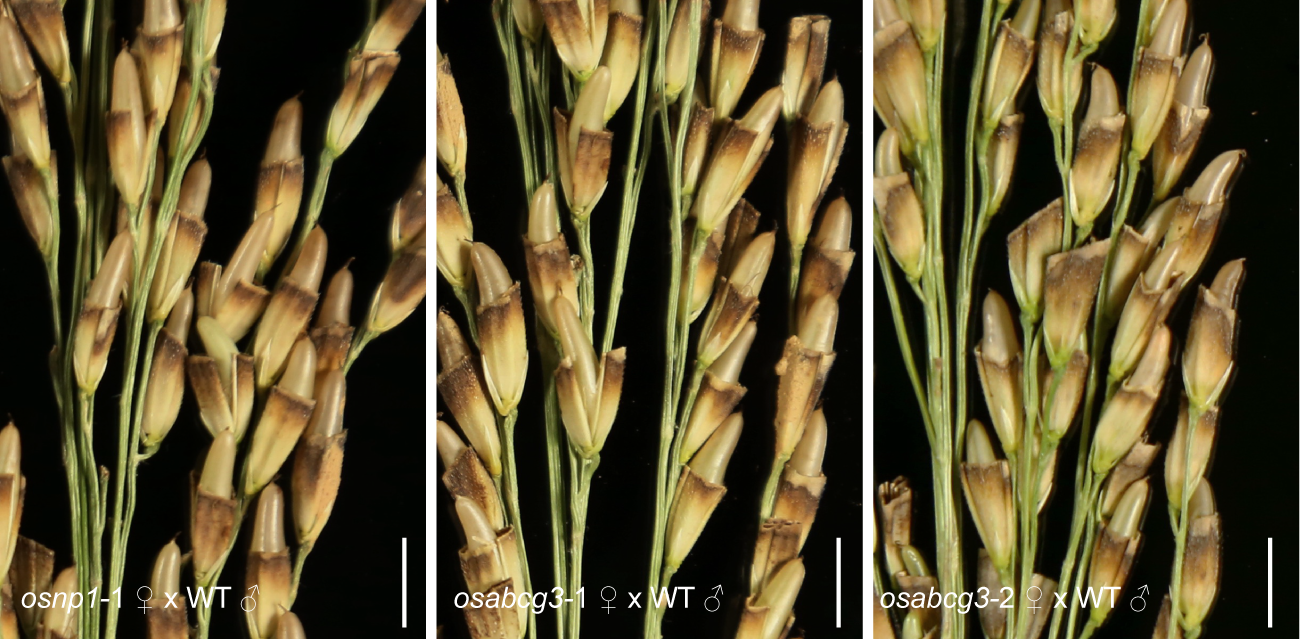

Supplement: Supplementary file 1 — Figure S1. Female fertility of osabcg3–1 and osabcg3–2 mutants. The seed set of osnp1–1, osabcg3–1 and osabcg3–2 after manually pollinated with WT pollen. Scale bars = 1 cm. (TIF 2498 kb) [file 12284_2018_248_MOESM1_ESM.tif]

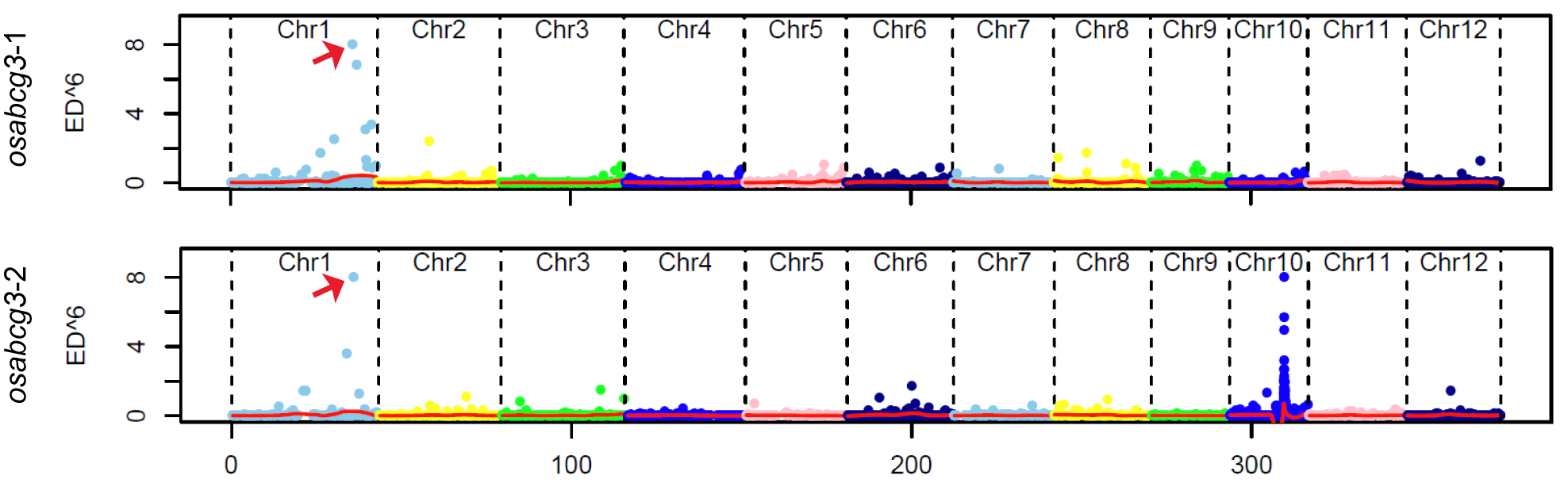

Supplement: Supplementary file 2 — Figure S2. Determination of the candidate mutation sites for osabcg3–1 and osabcg3–2. Loess curve was used to show the distribution of ED6 values of all SNPs along the chromosomes. The candidate regions harboring causal mutation were indicated by red arrows on chromosome 1. (TIF 282 kb) [file 12284_2018_248_MOESM2_ESM.tif]

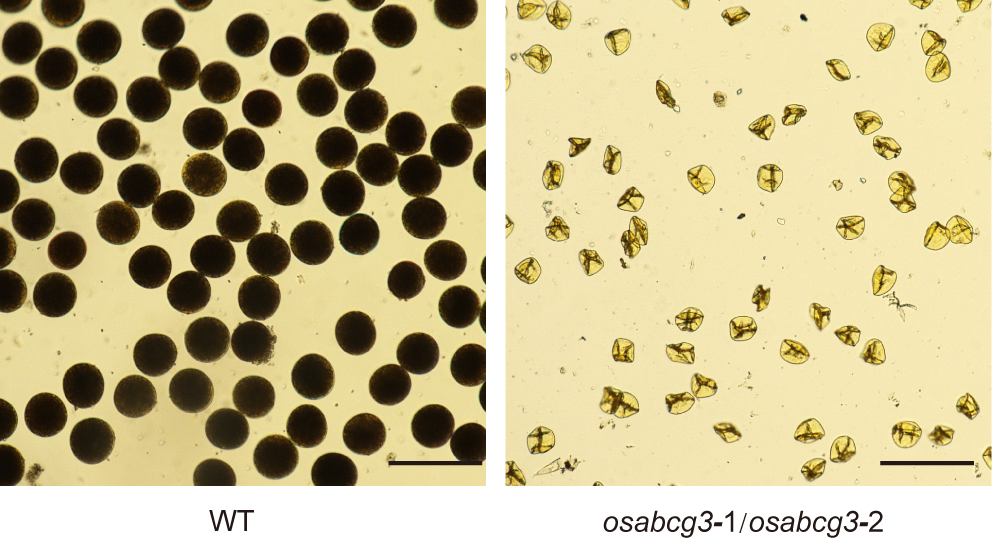

Supplement: Supplementary file 4 — Figure S3. Pollen grains of double heterozygote of osabcg3–1/osabcg3–2. Pollen grains of WT and osabcg3–1/osabcg3–2 double heterozygote were stained with I2-KI. Scale bars = 100 μm. (TIF 1208 kb) [file 12284_2018_248_MOESM4_ESM.tif]

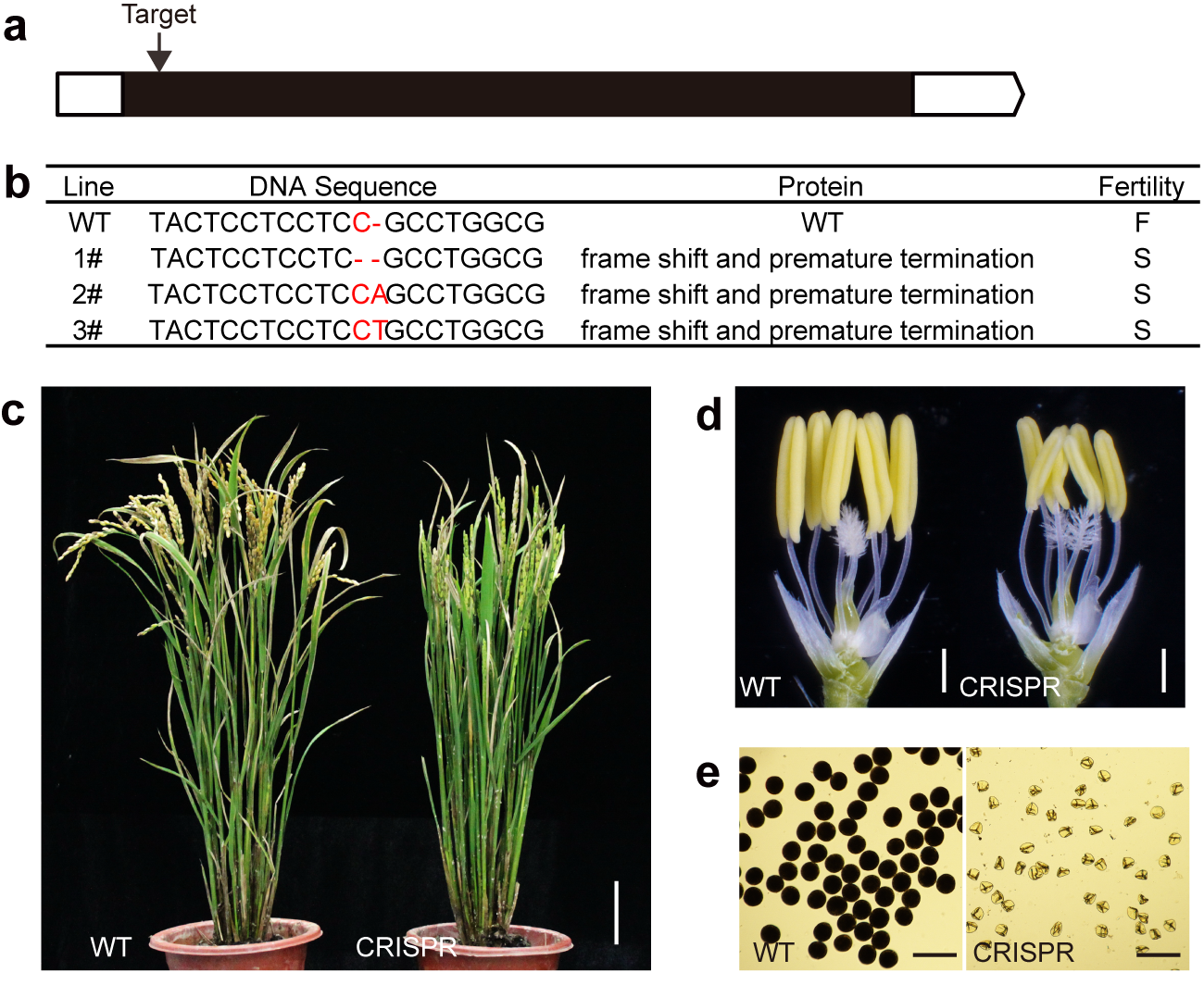

Supplement: Supplementary file 5 — Figure S4. Characterization of osabcg3 mutants in janponica Wuyungeng 7. a The site on OsABCG3 targeted by the CRISPR/Cas9 system. The arrowhead indicates the target site. b OsABCG3 sequence of three CRISPR-mutated lines in Wuyungeng 7 background. c-e Phenotype of WT and CRISPR-mutated plants. Plants at heading stage (c), spikelets with palea and lemma removed (d), and pollen grains stained with I2-KI (e) were showed. Scale bars = 10 cm (c); 1 mm (d); 100 μm (e). (TIF 1789 kb) [file 12284_2018_248_MOESM5_ESM.tif]

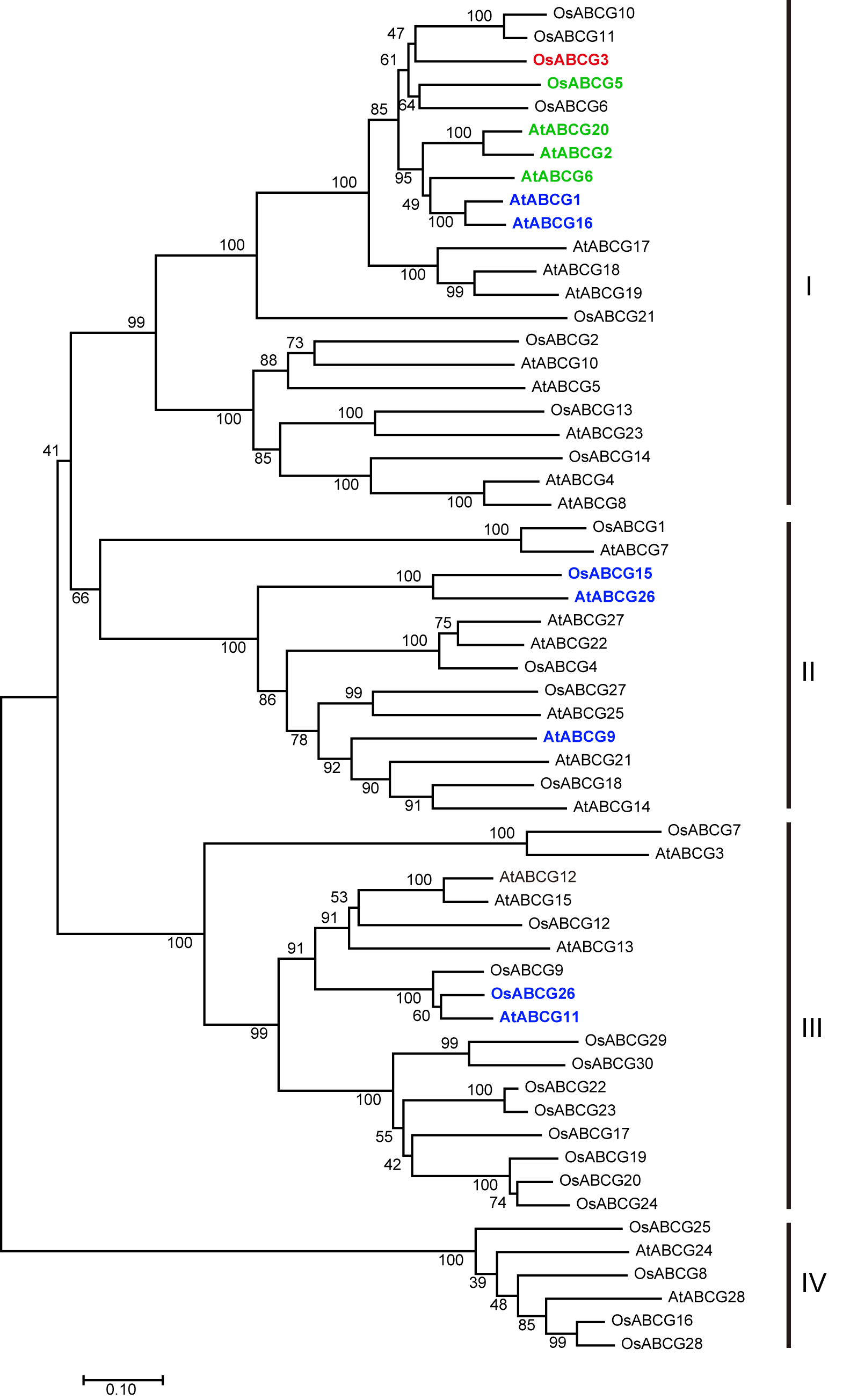

Supplement: Supplementary file 6 — Figure S5. Phylogenetic analysis of half-size ABCG proteins in rice and Arabidopsis. The half-size ABCG proteins in rice and Arabidopsis were aligned and used to construct a tree with MEGA7 software using the Neighbor-Joining method. OsABCG3 was marked in red, seven proteins required for pollen wall formation in blue, proteins for suberin formation in green, respectively. (TIF 562 kb) [file 12284_2018_248_MOESM6_ESM.tif]

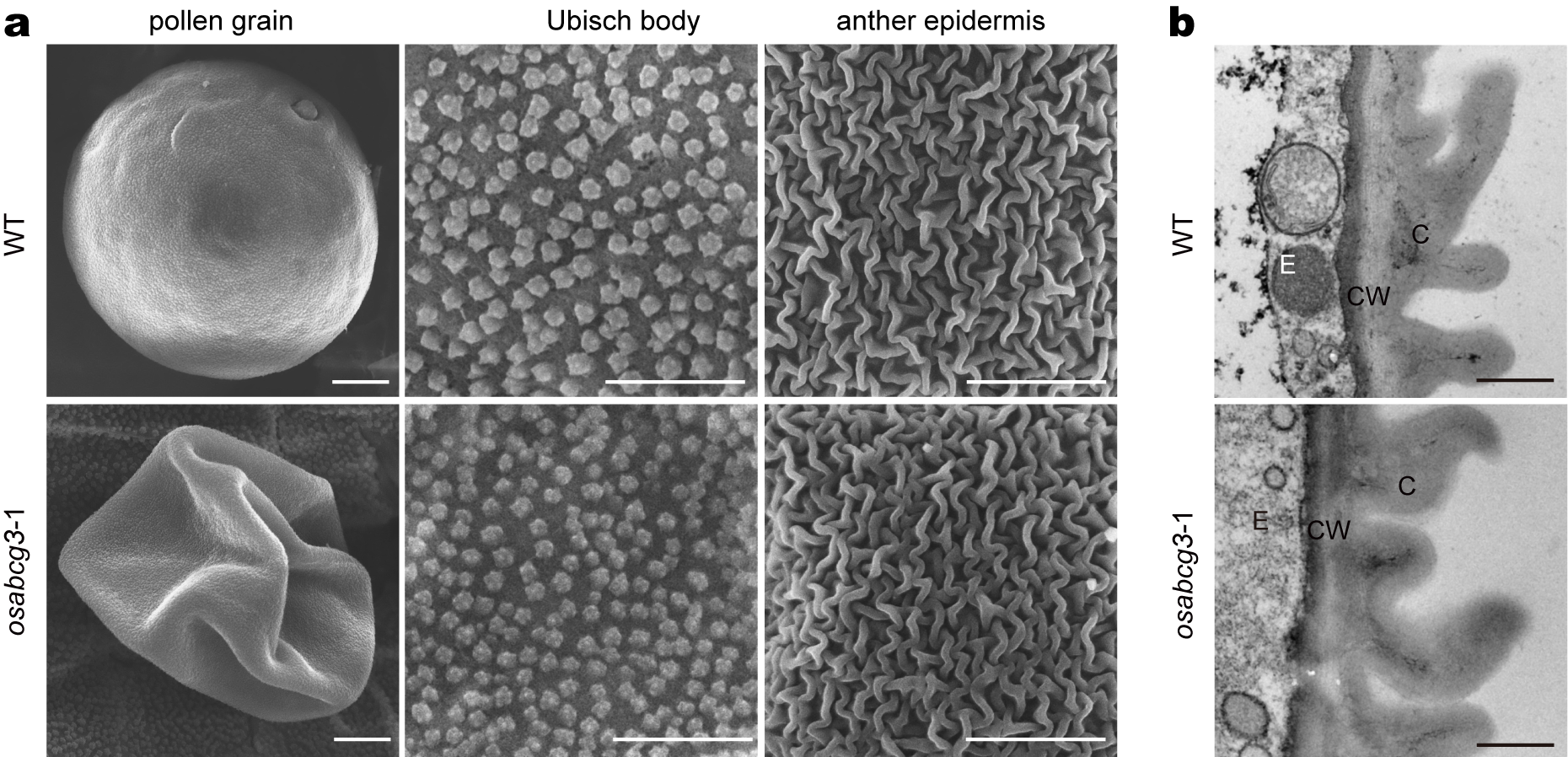

Supplement: Supplementary file 7 — Figure S6. SEM and TEM analysis of the pollen grain and anther surfaces at stage 12 in WT and osabcg3–1. a SEM analysis of the pollen grain, Ubisch body and anther epidermis in WT and osabcg3–1 at stage 12. b TEM observation of the anther cuticle at stage 12. Scale bars = 5 μm (a); 500 nm (b). (TIF 2237 kb) [file 12284_2018_248_MOESM7_ESM.tif]
