# Supplementary material for: The ATP-binding cassette (ABC) transporter OsABCG3 is essential for pollen development in rice
Source: Rice (N Y). 2018 Oct 11;11:58. doi: 10.1186/s12284-018-0248-8 (PMC6181869; doi:10.1186/s12284-018-0248-8)
Supplement: Supplementary file 3 — Table S1. SIMM analyses of osabcg3–1 and osabcg3–2 mutants. (DOC 41 kb) [file 12284_2018_248_MOESM3_ESM.doc]

**Table S1.** SIMM analyses of *osabcg3*-1 and *osabcg3*-2 mutants.

| Mutant | Total reads | Aligned reads | Aligned ratio (%) | Coverage (%) | Average depth | SNP^a^ | SNP^b^ | Candidate | Genotype |
| --- | --- | --- | --- | --- | --- | --- | --- | --- | --- |
| *osabcg3*-1 | 214,490,730 | 178,270,443 | 83.11 | 90.1 | 45× | 1,696,334 | 2,140 | Chr1: 35,863,242 | TGG:trp>TGA:stp |
| *osabcg3*-2 | 212,159,628 | 176,091,329 | 83 | 90.15 | 44× | 1,691,301 | 2,494 | Chr1: 35,862,929 | GGC:gly>GAC:asp |

^a^ Number of SNPs between the mutant and the Nipponbare reference genome (MSU7.0).

^b^ Number of EMS-induced SNPs identified with other HHZ mutants as backgrounds.
