# Supplementary material for: The ATP-binding cassette (ABC) transporter OsABCG3 is essential for pollen development in rice
Source: Rice (N Y). 2018 Oct 11;11:58. doi: 10.1186/s12284-018-0248-8 (PMC6181869; doi:10.1186/s12284-018-0248-8)
Supplement: Supplementary file 8 — Table S2. Primers used in this study. (DOC 56 kb) [file 12284_2018_248_MOESM8_ESM.doc]

**Table S2.** Primers used in this study

| Primer name | Forward (5′ to 3′) | Reverse (5′ to 3′) | Purpose |
| --- | --- | --- | --- |
| HRM-osabcg3-1 | CATCAACCGGGACAGGATAC | CGTTCTGGAGCACGCCCT | HRM analysis |
| HRM-osabcg3-2 | ACGAGCGCTACATCTTCCTC | AGAATGTGGTGAAGGCGAAG | HRM analysis |
| CR-OsABCG3 | GGCGGTGTTACTCCTCCTCCGCC | AAACGGCGGAGGAGGAGTAACACC | vector construction |
| SP-LR | GCGCGGTGTCATCTATGTTACT | CCCGACATAGATGCAATAACTTC | transgenic analysis |
| CR-OsABCG3-Seq | GAGATGCTCCGCCTCGTC | AGAATGTGGTGAAGGCGAAG | sequencing |
| Com-OsABCG3-P1 | CCTCTAGAGTCGACCTGCAGTTGGATGCGATACTCCCTCCG | CCAAGCTTGCATGCCTGCAGCCAAAATCCTAAACCTCTCGACT | vector construction |
| Com-OsABCG3-P2 | CACTGCCTTTCTTGTCTG | GCGATTAAGTTGGGTAACGC | transgenic analysis |
| Com-OsABCG3-P3 | CATGTCCACCATGTTCTACACCT | AAGGGGAGAGAAAGAGACAGAGA | background genotype |
| Pro-OsABCG3-P1 | ACTTCCCGGGGAATTCTTGGATGCGATACTCCCTCCG | ACTGCAGTGGGAATTCGGCATGCACACCCACAGCTA | vector construction |
| Pro-OsABCG3-P2 | GCAACTGGACAAGGCACTAGCG | CGGTTCAGGCACAGCACATCAA | transgenic analysis |
| CDs-OsABCG3 | CCGGAATTCTATGGTCCTAACCACACCTAGGAC | CGCGGATCCTCACCTCCTCTTGTTCTTGC | vector construction |
| CDs-OsRac3 | GCATGGACGAGCTGTACAAGATGGCGTCCAGCGCCTCCCGGTTC | GAAATTCGAGCTTCTCGAGTTATTAGGATTTGAAGCATGAC | vector construction |
| qPCR-OsABCG3 | CACGTCATGATCGGCTACAC | ACGAGCGAGAGGTAGTGGAA | qRT-PCR |
| qPCR-OsABCG26 | GGCTGATCACCTACTACAAGAACTC | CTCCTGCATCCAGCACTGTC | qRT-PCR |
| qPCR-OsABCG15 | CATGTGTGGCCAACCAAAGA | GTGGTCTTGCCACTGCCAGA | qRT-PCR |
| qPCR-TDR | TGCTCTGGGAGCACAAGCC | CTCGCTGTCCCTCACCATG | qRT-PCR |
| qPCR-PTC1 | CACCAGATCATGGACCTCTG | AGCAGCCTCAGCTCCATGTG | qRT-PCR |
| qPCR-WDA1 | ACCACTCTTCAATCGTCACTGAG | GCAGTTCCAGTCAAGGCACAG | qRT-PCR |
| qPCR-CYP704B2 | GCTGGTTGATGACTTCACCT | CGACAGTATGTCGTGCTTGAT | qRT-PCR |
| qPCR-DPW2 | CCAGGGTCCTTGTCCACTAC | CACGATAAGCTTCCCGTCGT | qRT-PCR |
| qPCR-OsTEK | CGTTGGAGGACGACGAGCTA | AGTCCTTGGAAGAGGCTTGG | qRT-PCR |
| qPCR-OsC4 | TGCCTAAGACGAGACGAGAG | GCCAAAGGAGGTCATCGTTA | qRT-PCR |
| qPCR-OsC6 | CTCCATCTGCCTGAGTATAT | GTCCATGCATGTTGCAGAAT | qRT-PCR |
| qPCR-OsACTIN1 | GCTATGTACGTCGCCATCCA | GGACAGTGTGGCTGACACCAT | qRT-PCR |
| ISH-OsABCG3-S | TAATACGACTCACTATAGGGAGAATGGCGCGCGCAGTG | CCATGGCCTCGCGCGGGACGA | ISH |
| ISH-OsABCG3-AS | TAATACGACTCACTATAGGGAGACCTCGCGCGGGACGAGT | ATGGCGCGCGCAGTG | ISH |
